# The Role of microRNAs in Cancer: Functions, Biomarkers and Therapeutics

**DOI:** 10.3390/cancers14040872

**Published:** 2022-02-10

**Authors:** Paola Tucci

**Affiliations:** Department of Pharmacy, Health and Nutritional Sciences, University of Calabria, 87036 Rende, Italy; paola.tucci@unical.it; Tel.: +39-0984493185

MicroRNAs (miRs) are small non-coding RNAs acting as post-transcriptional regulators of gene expression with important roles in almost all biological pathways, including development, differentiation, cell cycle, proliferation, and apoptosis. Deregulated miR expression has been detected in numerous cancers, where miRs act as both oncogene and tumor suppressors. Considering their important roles in tumorigenesis, miRs have been investigated as prognostic and diagnostic biomarkers and as useful targets for therapeutic intervention.

This Special Issue of *Cancers* focuses on the identification and characterization of new miR targets involved in cancer pathogenesis and on their role as potential biomarkers for early detection and diagnosis, as well as for the development of new cancer therapy. Some more recent and exciting advances in the field are collected and presented here, providing new ideas for discussion of future perspectives among researchers working on this hot topic.

The review by Katsaraki et al. [1] summarizes and highlights the current knowledge concerning the multifaceted role of miRs both in normal B-cell development and B-cell chronic lymphocytic leukemia progression, prognosis and therapy.

Recent studies revealed differences in the miR expression profiles in tissues from patients with ovarian cancer and healthy individuals. For example, the expression of miR-200a, miR-200b, and miR-200c was significantly higher than that in normal tissues, whereas mir-199a, miR-140, miR-145, and miR-125b1 displayed low expression in ovarian cancer tissues. A potential explanation for a global decrease in miR expression may be inhibition of DICER, as shown by Wilczynski et al. [2]. Down-regulation of DICER has been detected in epithelial ovarian cancer and was associated with the up-regulation of the oncogenic miRNA-103/107. Although the results of their study do not highlight any clinical or prognostic role of the miRs, the miR-103/miR-107/DICER axis may be one of the key regulators of cancer aggressiveness [2].

In their paper, Jasinski-Bergner et al. [3] describe for the first time the impact and relevance of factors involved in 2′-O-methylation and pseudouridylation of different RNA species, such as miRs, related to the processes of tumor formation and progression in a malignant melanoma model. The up-regulation of the RNA modifying proteins is, indeed, a prognostic factor in this tumor and the impact of these molecules on miRs would lead to the identification of new proteins involved in the miRs deregulation, thus suggesting that both RNA factors and miRs involved in this process, represent suitable targets for tumor therapy and putative novel prognostic markers [3].

MiRs are not only able to distinguish normal tissues from tumor, but can also distinguish and characterize the different tumoral subgroups. A few studies have dedicated their attention to the detection and monitoring of renal cancer, one of the most common cancers worldwide with a nearly non-symptomatic course until the advanced stages of the disease. Kajdasz et al. [4], through a meta-analysis study, investigate and validate the changes in miRs expression in renal cancer patients. Furthermore, deregulated miRs are differentially expressed in the different renal cancer subtypes and can be used to distinguish the subclass [4].

In case of medulloblastoma, the most common malignant brain tumor in children, studies have mostly concentrated on the clinical entity of the single disease rather than in the four molecular subgroups [5]. Each subgroup has a different cell of origin, prognosis, and may require specific therapeutic strategies. The review by Bevacqua et al. [5] summarizes the role of miRs in the four medulloblastoma subgroups, their potential as biomarkers for early diagnosis and prognosis, highlighting the potential of these miRs in providing new opportunities to treat the different clinical and biological features between subgroups.

The role of various miRs was investigated in relation to metabolic disorders, as suggested by Sidorkiewicz et al., showing the alterations in miR profiles in endometrial cancer patients with insulin resistance [6].

On the other hand, another study by Lee et al. [7] links the expression of miRs to the effects of cigarette smoking, a major risk factor of lung cancer by inducing DNA methylation. Regarding this, the authors found that miR-584-5p expression was down-regulated by the methylation and this resulted in increased migration and invasion in smoking-related lung cancer cells by targeting the oncogenic protein YKT6 [7]. Thus, the tumor suppressor miR-584-5p might be used as molecular biomarker for this kind of cancer.

One of the factors contributing to the complexity of tumor growth, metastasis, and patient survival in breast cancer is the level of hypoxia (oxygen deficiency) [8]. To counteract hypoxia, cancerous cells secrete growth factors that facilitate angiogenesis in the tumor microenvironment to deliver the required oxygen and nutrients to tumoral cells, as well as oxidative stress, epithelial to mesenchymal transition, cell migration, and inflammation in cancer. Gervin et al. [8] identify and investigates a novel function of miR-526b and miR-655 in breast cancer, showing that hypoxia enhances oncogenic functions of these miRs in breast cancer cells and promotes the expression of tumor-associated angiogenic marker and tumoral progression.

During carcinogenesis, miRs play important roles in regulating the maintenance and acquisition of cancer stem cells. Two papers discuss on this issue [9,10]. In the first paper, Liao et al. [9] identify in colorectal stem-like cancer cells the microRNA-210-Stathmin1 axis, critical for inducing microtubule destabilization, decreasing cell elasticity and thus facilitate cell motility and metastasis. In the second, Fitriana at al. [10] summarize the latest finding on the role of miRs in regulating cancer stemness with regard to the head and neck cancers, and analyzed them as useful targets for potential clinical application. 

As tumor cells can release miRs resistant to digestion by RNases through their encapsulation into microvesicles or binding to lipoproteins, the use of circulating miRs as biomarkers for different cancer types is a rapidly developing area. miRs can be detected in biological fluids, allowing non-invasive diagnosis to discriminate malignant lesions from benign lesions. Gajek et al. [11] summarize the latest findings on the utility of miRs as potential biomarkers for ovarian cancer diagnosis and prognosis as circulating miR profiles reflect the tumor profiles. Furthermore, by modulating the sensitivity of the cancer cells to chemotherapeutic agents they might serve as promising therapeutic for multidrug-resistance ovarian cancer [11].

Giussani et al. [12] analyze circulating miRs in plasma sample from patients enrolled in the clinical study and identified 5 miRs (miR-625, miR-423-5p, miR-370-3p, miR-181c, and miR-301b) that, properly combined, are able to distinguish malignant from benign breast disease in women [12].

Since recent studies have shown that exosomes promote the generation of a metastatic niche by transferring functional molecules, Eun et al. [13] investigated the role of circulating exosomal miRs in cancer metastasis, and found that the exo-miR-1307-5p was significantly overexpressed in hepatocellular carcinoma and correlated with progression and metastasis in patients with advanced-stage. For a precision treatment strategy, the identification of metastasis driver molecules in blood would help classify patients in accordance with the risk of metastasis during the initial staging process [13]. 

Finding biomarkers for metastasis is also important for identifying melanoma tumors. The aim of the pilot study by Bustos et al. [14] was to demonstrate the utility of circulating cell-free miRs as potential blood biomarkers for stage III and IV melanoma patients compared to serum lactate dehydrogenase which is currently an accepted biomarker for stage IV, but it has limited utility for stage III melanoma patients. Thus, they identified several miRs suitable for real-time monitoring treatment response of patients with metastatic melanoma [14].

In summary, this Special Issue of *Cancers* is a collection of articles (nine research articles and five reviews articles) discussing the role of miRs in cancer. The identification and characterization of new cancer-relevant miRs may be used to facilitate patient diagnosis and prognosis of different tumors. Moreover, miR profiles can define relevant tumoral subtypes. We can also monitor miR changes to predict therapeutic responses as a non-invasive detection method. Lastly, the importance of miRs in cancer has paved the way for new diagnostic and therapeutic opportunities. Even though substantial questions must be answered, with the advances in in vivo delivery systems, the administration of miR-based therapeutics is feasible and safe in humans, and they could represent a suitable target for the clinical treatment, able to change the medical practice in the foreseeable future.
